# Gluten-Free Foods Cooked in Shared Fryers With Wheat: A Pilot Study Assessing Gluten Cross Contact

**DOI:** 10.3389/fnut.2021.652039

**Published:** 2021-03-23

**Authors:** Tricia Thompson, Trisha Bury Lyons, Amy Keller, Nancee Jaffe, Luke Emerson-Mason

**Affiliations:** ^1^Gluten Free Watchdog, LLC, Manchester, MA, United States; ^2^Department of Clinical Nutrition, MetroHealth Medical Center, Cleveland, OH, United States; ^3^Nutrition Services, Mary Rutan Hospital, Bellefontaine, OH, United States; ^4^UCLA Digestive Health & Nutrition Clinic, University of California, Los Angeles, Los Angeles, CA, United States; ^5^Bia Diagnostics, LLC, Colchester, VT, United States

**Keywords:** gluten, wheat, cross contact, shared fryers, competitive R5 ELISA

## Abstract

**Introduction:** Consumers with celiac disease are discouraged from eating fried foods cooked in shared fryers with wheat-containing foods at restaurants based on presumed gluten exposure. The purpose of the present study is to assess gluten levels of fries free of gluten-containing ingredients cooked in shared fryers with wheat.

**Methods:** 20 orders of fries were purchased from 10 different restaurants. Restaurants confirmed that fries and oil were free of gluten-containing ingredients. All restaurants confirmed that their fryers were used to cook wheat-containing foods. Fries were sent to Bia Diagnostics and tested in 1-gram duplicates using the R7001 sandwich R5 ELISA and the R7021 competitive R5 ELISA. A microwave control also was run.

**Results:** The sandwich ELISA found gluten in 9/20 fry orders (7 to > 80 ppm). The competitive ELISA found gluten in 3/20 fry orders (14 to > 270 ppm). In the microwave control (60-ppm gluten mixture of wheat flour and canola oil), the unheated mixture tested at a mean level of 64 ppm gluten using the sandwich ELISA and 137 ppm gluten using the competitive ELISA. The mixture heated to 190°C tested at a mean level of 55 ppm gluten using the sandwich ELISA and < 10 ppm and 16 ppm gluten using the competitive ELISA.

**Discussion:** Based on test results, 25% of fry orders would not be considered gluten-free.

**Summary:** Gluten cross contact may occur when gluten-free foods are cooked in shared fryers with wheat. ELISAs may underperform when analyzing for gluten that has been heated.

## Introduction

Dietitians have long been discouraging consumers with celiac disease (CD) from ordering gluten-free foods cooked in the same deep fryer as gluten-containing foods at restaurants. This recommendation is based on presumed gluten exposure vs. evidence-based research that gluten cross contact occurs. To the best of the authors' knowledge there is no published data on gluten levels of gluten-free foods after cooking in shared fryers. The lack of evidence of cross contact contributes to confusion among consumers, especially when gluten-free foods cooked in shared fryers (e.g., fries) are marked as gluten-free on some restaurant menus. The purpose of the present study is to help inform consumer recommendations by assessing gluten levels of fries free of gluten-containing ingredients cooked in shared fryers with wheat.

## Methods

A convenience sample of 20 orders of fries was purchased from 10 different restaurants in California and Ohio. Prior to purchase, restaurants confirmed that fries and oil were free of gluten-containing ingredients. Restaurants also were asked specifically if their fries or oil contained any wheat, malt or gluten ingredients. Fries were ordered plain with salt only. All restaurants confirmed that their fryers were used to cook wheat-containing products (e.g., fried chicken/fish, onion rings, fried sandwiches). Because the gluten level in a shared fryer may vary, two separate orders of fries were purchased from each restaurant on consecutive Saturday afternoons.

Each order of fries was placed unopened into a coded bag. Fries were mailed to Bia Diagnostics, LLC, Colchester, VT, USA (ISO Accredited Lab). Each individual order of fries was homogenized using a blender and tested in 1-gram duplicates using the Ridascreen Gliadin R7001 sandwich R5 enzyme-linked immunosorbent assay (ELISA) and extracted with the cocktail solution (Art. No. R7006) following the kit manufacturer's directions (R-biopharm, Darmstadt, Germany) (1). Samples were also tested in 1-gram duplicates using the Ridascreen Gliadin R7021 competitive R5 ELISA and extracted with ethanol following the kit manufacturer's directions (2). A total of 80 extractions were tested (4 extractions from each sample).

To assess whether the sandwich and competitive ELISAs are fit for purpose to test for the presence of gluten in products heated in oil, a microwave control was run. A 60 mg/kg (ppm) gluten mixture of wheat flour and canola oil was prepared by Bia Diagnostics and tested for gluten before and after heating in a microwave to 190°C/374°F (within temperature range recommended by the U.S. Department of Agriculture for deep frying chicken) (3). Samples were tested in duplicate using the sandwich and competitive R5 ELISAs.

## Results

### Fries

The sandwich R5 ELISA found quantifiable levels of gluten in 9 of 20 (45%) orders of fries ranging from 7 to > 80 mg/kg (ppm) (above the highest standard) (Table 1). Five orders (25%) of fries tested above 20 mg/kg (ppm) of gluten. Fries from 6 of the 10 (60%) restaurants were found to contain quantifiable levels of gluten in at least 1 of the 2 orders, with fries from 4 of these 6 restaurants found to contain levels above 20 mg/kg (ppm) of gluten in at least 1 of the 2 orders. The competitive R5 ELISA found gluten in 3 of the 20 (15%) orders of fries ranging from 14 to > 270 mg/kg (ppm) gluten (above the highest standard).

**Table 1:** Gluten levels mg/kg (ppm) in samples tested.

**Gluten levels of restaurant fries cooked in shared fryers with wheat-containing foods**					
**Test code**	**Sample tested**	Sandwich^*^ Extraction 1	**Sandwich** **Extraction 2**	Competitive^**^ **Extraction 1**	**Competitive** **Extraction 2**
F1A	Plain fries, salt only	<5	<5	<10	<10
F1B	Plain fries, salt only	<5	<5	<10	<10
F2A	Plain fries, salt only	<5	<5	<10	<10
F2B	Plain fries, salt only	18	19	<10	<10
F3A	Plain fries, salt only	45	28	19	14
F3B	Plain fries, salt only	52	62	29	31
F4A	Plain fries, salt only	<5	<5	<10	<10
F4B	Plain fries, salt only	<5	<5	<10	<10
F5A	Plain fries, salt only	11	7	<10	<10
F5B	Plain fries, salt only	11	9	<10	<10
F6A	Plain fries, salt only	<5	<5	<10	<10
F6B	Plain fries, salt only	<5	<5	<10	<10
F7A	Plain fries, salt only	19	15	<10	<10
F7B	Plain fries, salt only	65	> 80	> 270	> 270
F8A	Plain fries, salt only	28	23	<10	<10
F8B	Plain fries, salt only	<5	<5	<10	<10
F9A	Plain fries, salt only	<5	<5	<10	<10
F9B	Plain fries, salt only	<5	<5	<10	<10
F10A	Plain fries, salt only	<5	<5	<10	<10
F10B	Plain fries, salt only	24	22	<10	<10
**Gluten levels mg/kg (ppm) of wheat flour and oil mixture in microwave control**					
**Temp**.	**Sample tested**	**Sandwich** **Extraction 1**	**Sandwich** **Extraction 2**	Competitive^***^ **Extraction 1**	**Competitive** **Extraction 2**
Unheated	Wheat flour & oil mixture (60 mg/kg)	72	55	165	109
Heated to 190°C/374°F	Wheat flour & oil mixture (60 mg/kg)	49	60	16	<10

* *The lower limit of quantification for the sandwich R5 ELISA is 5 mg/kg (ppm) of gluten. The R7001 assay is a Codex Alimentarius Type 1 Method and an AOAC Official Method of Analysis (1). It is also one of two assays that FDA has stated they will use if testing is necessary as part of gluten-free rule enforcement (4)*.

** *The lower limit of quantification for the competitive R5 ELISA is 10 mg/kg (ppm) of gluten. The R7021 is an AOAC Official First Action Method (2). Gluten protein fragments cannot be adequately detected using a sandwich ELISA. When gluten protein fragments are suspected, a competitive ELISA is recommended*.

*** *In the microwave control, the results of the unheated sample are overestimates when assessed using the competitive ELISA. The competitive ELISA is intended to analyze the presence of protein fragments. Generally, results using the competitive will be higher as compared to the sandwich when assessing intact gluten. The competitive ELISA requires only a single epitope to detect gluten while a sandwich ELISA requires two (2)*.

### Microwave Control

The unheated oil and wheat flour mixture tested at a mean level of 64 mg/kg (ppm) of gluten using the sandwich R5 ELISA and 137 mg/kg (ppm) of gluten using the competitive R5 ELISA (Table 1). The oil and wheat flour mixture heated to 190°C/374°F tested at a mean level of 55 mg/kg (ppm) of gluten using the sandwich R5 ELISA and < 10 mg/kg (ppm) and 16 mg/kg (ppm) of gluten using the competitive R5 ELISA.

## Discussion

Testing found varying levels of gluten in the fry samples, including samples tested from the same restaurant. The gluten level in a shared fryer at any given time likely varies depending upon several factors, including previously cooked foods, oil change frequency, and filtration system. The impact of these factors on the gluten level in fryer oil is worthy of further research.

While orders were placed only with restaurants confirming that fries were free of gluten-containing ingredients, it was not feasible given the real world nature of this study to verify gluten-free status by testing uncooked fries. However, a study on gluten levels of packaged foods not labeled gluten-free but appearing to be free of gluten containing ingredients, found that <5% contained levels of gluten at or above 20 mg/kg (ppm) (5). While it is possible that some gluten present in the tested fries could have been from the uncooked fries themselves vs. cross contact due to the presence of wheat in the shared oil, this seems relatively unlikely. In future studies, it would be useful to partner with restaurants to test raw ingredients in addition to testing finished food products.

A microwave vs. a deep fryer was used for the control. Using a fryer in the lab proved challenging due to difficulty in maintaining a homogeneous flour and oil mixture, preventing precipitation of the flour, and preventing burning of the flour on the heating element. This was true even when the flour and oil mixture was placed in a beaker.

**Figure 1:**
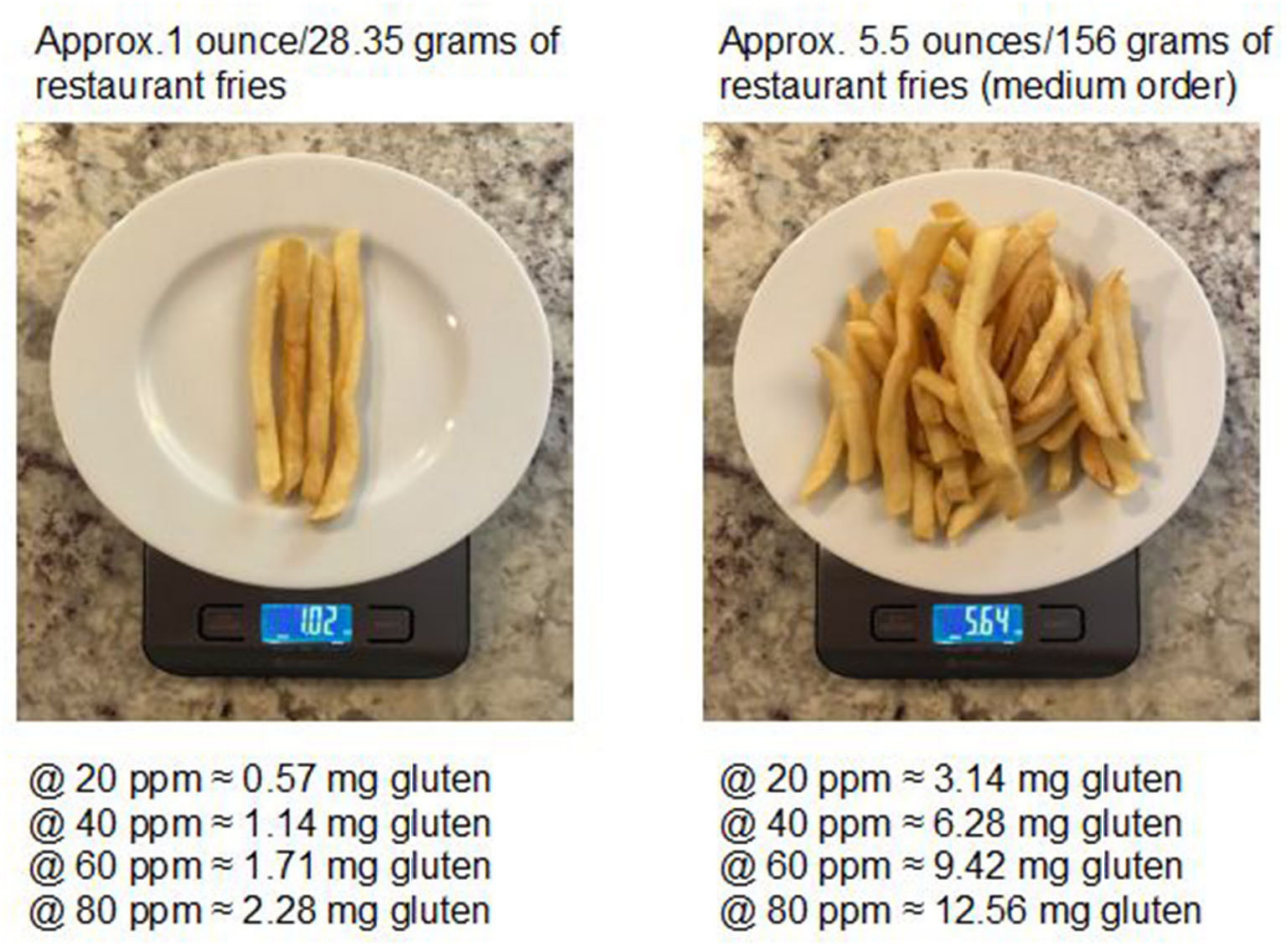
Milligram amount of gluten in restaurant fries at various ppm levels^*^, ^**^. ^*^ Each 1 ounce/28.35 g portion of fries at a gluten level of 20 ppm contains 0.57 mg of gluten.^**^ 10 mg of gluten per day is considered by experts to be a tolerable amount for most individuals with celiac disease (6).

Based on test results, 5 of the 20 (25%) orders of fries would not be considered gluten-free (4); 15 (75%) of the fry orders would be considered gluten-free. Gluten cross contact in fries may add substantial amounts of gluten to the diet, depending upon the amount of fries consumed (Figure 1).

It may be the case that all ELISAs underperform when analyzing for gluten that has been heated (7). This may be due to a decrease in solubility of the gluten (i.e., ability of gluten to dissolve in solution to be extracted) as a result of exposure to high temperatures (8). Increased temperatures also may result in denaturation (i.e., change in structure) of the gluten present in samples, reducing their affinity to the antibodies used in the ELISA methods (9). The impact of processing, including heating, on gluten has yet to be fully elucidated.

Results using the sandwich R5 ELISA may underestimate gluten levels in the cooked fries (1, 10). According to R-Biopharm, “In processed food (e.g., heat treatment, dehydration, etc.), proteins may be altered or fragmented, this may have an impact on the recovery/cross reactivity” (1). Results using the competitive R5 ELISA also may underestimate gluten levels in the cooked fries for the same reason (2, 10). In the microwave control, the gluten level appeared to fall to almost unquantifiable levels when the mixture was heated to 190°C/374°F as compared to the unheated sample. According to R-Biopharm, “Heat treated samples that are extracted with ethanol show a reduced recovery” (2). Ethanol is the extraction solution used with the competitive ELISA (2). For this reason, R-Biopharm recommends that heat treated samples be extracted with the cocktail solution and analyzed with the sandwich ELISA (2). However, the sandwich R5 ELISA is not recommended for foods when gluten proteins may have become fragmented due to processing (1). There is a need for improved analytical methods for gluten analysis to address foods that may be both heat treated and contain fragmented gluten.

The impact of heat on the ability of ELISAs to accurately detect and quantify gluten is an area that requires additional research. As pointed out by Panda and Garber, the limitations of ELISAs are further compounded by the lack of clinical information regarding the immunopathogenicity of gluten peptide fragments as compared to intact gluten protein (10). While the solubility, fragmentation, or denaturation of gluten may impact the ability of ELISAs to accurately detect and quantify it, this doesn't mean that gluten is rendered “safe” for persons with CD. As stated by Sharma et al., while assays may underestimate gluten content in processed foods due to incomplete extraction, this does not mean gluten isn't present in amounts deemed unsafe for consumers (9).

## Summary

Results of this assessment suggest that gluten cross contact may occur when gluten-free foods are cooked in shared fryers with wheat. While a much larger study may be warranted, it remains prudent to advise consumers with CD to avoid foods cooked in shared fryers. It is impossible for a consumer to know how much gluten is in fryer oil and how much gluten may end up in an order of fries. Shared holding trays, scoops, and fryer baskets also are sources of potential cross contact. The gluten levels reported in this investigation may be underestimates due to the limitations of the analytical methods available for gluten analysis of foods heated to high temperatures.

## Data Availability Statement

The original contributions presented in the study are included in the article/supplementary material, further inquiries can be directed to the corresponding author/s.

## Author Contributions

LE-M performed the laboratory analysis. TT wrote the first draft of the manuscript. All authors contributed to the conception, design of the study, analysis of the data, manuscript revision, read, and approved the submitted version.

## Conflict of Interest

TT is the owner and founder of Gluten Free Watchdog, LLC. LE-M is an employee of Bia Diagnostics, LLC. The remaining authors declare that the research was conducted in the absence of any commercial or financial relationships that could be construed as a potential conflict of interest.
